# Data Resource Profile: The Korea National Health and Nutrition Examination Survey (KNHANES)

**DOI:** 10.1093/ije/dyt228

**Published:** 2014-02-27

**Authors:** Sanghui Kweon, Yuna Kim, Myoung-jin Jang, Yoonjung Kim, Kirang Kim, Sunhye Choi, Chaemin Chun, Young-Ho Khang, Kyungwon Oh

**Affiliations:** ^1^Division of Health and Nutrition Survey, Korea Centers for Disease Control and Prevention, Cheongwon-gun, Korea, ^2^Department of Preventive Medicine, University of Ulsan College of Medicine, Seoul, Korea and ^3^Institute of Health Policy and Management, Seoul National University College of Medicine, Seoul, Korea

## Abstract

The Korea National Health and Nutrition Examination Survey (KNHANES) is a national surveillance system that has been assessing the health and nutritional status of Koreans since 1998. Based on the National Health Promotion Act, the surveys have been conducted by the Korea Centers for Disease Control and Prevention (KCDC). This nationally representative cross-sectional survey includes approximately 10 000 individuals each year as a survey sample and collects information on socioeconomic status, health-related behaviours, quality of life, healthcare utilization, anthropometric measures, biochemical and clinical profiles for non-communicable diseases and dietary intakes with three component surveys: health interview, health examination and nutrition survey. The health interview and health examination are conducted by trained staff members, including physicians, medical technicians and health interviewers, at a mobile examination centre, and dieticians’ visits to the homes of the study participants are followed up. KNHANES provides statistics for health-related policies in Korea, which also serve as the research infrastructure for studies on risk factors and diseases by supporting over 500 publications. KCDC has also supported researchers in Korea by providing annual workshops for data users. KCDC has published the *Korea Health Statistics* each year, and microdata are publicly available through the KNHANES website (http://knhanes.cdc.go.kr).

## Data resource basics

KNHANES is an ongoing surveillance system in the Republic of Korea (hereafter ‘Korea’) that assesses the health and nutritional status of Koreans, monitors trends in health risk factors and the prevalence of major chronic diseases and provides data for the development and evaluation of health policies and programmes in Korea.^1^ KNHANES was first established in 1998 based on the Article 16 of the National Health Promotion Act proclaimed in 1995. This surveillance system has been conducted by KCDC. KNHANES is a nationwide cross-sectional survey conducted every year, and its target population comprises nationally representative non-institutionalised civilians in Korea. Each survey year includes a new sample of about 10 000 individuals aged 1 year and over.

**Figure 1:**
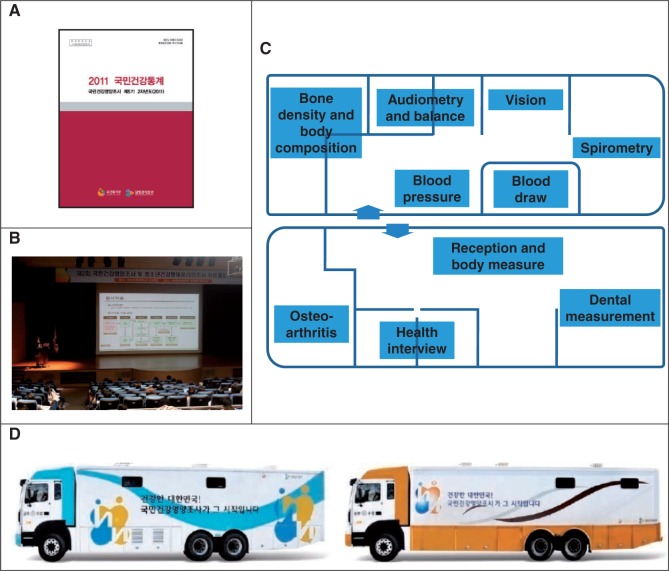
(A) The Korea Health Statistics, the official report of the Korea National Health and Nutrition Examination Survey (KNHANES) by the Ministry of Health and Welfare and the Korea Centers for Disease Control and Prevention (KCDC). (B) The workshop for KNHANES data users provided by KCDC. (C) Survey in the mobile examination centre (MEC) of KNHANES. (D) One unit of MEC (two trucks)

KNHANES is composed of three component surveys: a health interview, health examination and nutrition survey. The health interview and health examination are performed by trained medical staff and interviewers at the mobile examination centre (MEC, see (C) and (D) in Figure 1). One week after the health interview and health examination surveys, dieticians visit the homes of participants for the nutrition survey. The surveys collect detailed information on socioeconomic status, health behaviours, quality of life, healthcare utilization, anthropometric measures, biochemical profiles using fasting blood serum and urine, measures for dental health, vision, hearing and bone density, X-ray test results, food intake and dietary behaviour.

## Data resource area and population coverage

The target population of KNHANES comprises non-institutionalized Korean citizens residing in Korea. The sampling plan follows a multi-stage clustered probability design. For example, in the 2011 survey, 192 primary sampling units (PSUs) were drawn from approximately 200 000 geographically defined PSUs for the whole country.^2^ A PSU consisted of an average of 60 households, and 20 final target households were sampled for each PSU using systematic sampling; in the selected households, individuals aged 1 year and over were targeted. The number of participants is shown in Table 1. The numbers of participants of the first three surveys (1998, 2001 and 2005) were approximately 35 000 in each survey. From 2007 the survey became a continuous programme with about 10 000 individuals each year except for the year 2007, when the number of participants was half of that of other years as the 2007 survey was conducted during a half-year (from July through December). All statistics of this survey have been calculated using sample weights assigned to sample participants.

**Table 1:** Characteristics of the Korea National Health and Nutrition Examination Survey (KNHANES) participants

Classification	KNHANES I (1998)	KNHANES II (2001)	KNHANES III (2005)	KNHANES IV			KNHANES V	
				1^st^ year (2007)^a^	2^nd^ year (2008)	3^rd^ year (2009)	1^st^ year (2010)	2^nd^ year (2011)
1 year and older total	38 551	37 434	33 805	4594	9744	10 533	8958	8518
								
Sex								
Male	18 770	18 241	16 167	2097	4370	4843	4115	3867
Female	19 781	19 193	17 638	2497	5374	5690	4843	4651
								
Age (years)								
1–9	5178	5079	3943	671	1324	1302	1142	1010
10–18	5628	4955	4375	588	1242	1338	1076	942
19–29	6138	5791	4641	407	960	1137	846	755
30–39	6808	6673	5582	728	1515	1508	1342	1204
40–49	5549	6243	5868	636	1403	1583	1279	1163
50–59	4017	3728	3975	549	1192	1302	1245	1265
60–69	3212	3032	3217	529	1118	1250	1081	1090
70+	2021	1933	2204	486	990	1113	947	1089
								
Area of residence								
Urban area	25 511	30 105	27 621	3328	7442	8088	7139	6947
Rural area	13 040	7329	6184	1266	2302	2445	1819	1571

^a^From 2007 the survey became a continuous programme with about 10 000 individuals each year except for the year 2007, when the number of participants was half that of the other years as the survey was conducted during a half-year (from July through December).

The sample weights were constructed for sample participants to represent the Korean population by accounting for the complex survey design, survey non-response and post-stratification. The weights based on the inverse of selection probabilities and inverse of response rates were modified by adjusting them to the sex- and age-specific Korean populations (post-stratification).

## Survey frequency and response rate

KNHANES has been conducted in 1998, 2001, 2005, 2007–09 and 2010–12. In 2013, we are now starting the sixth KNHANES (2013–15). The first and second surveys were performed in November and December of 1998 and 2001, and the third survey was conducted from April to June of 2005. Since then, the frequency of KNHANES has been redesigned from once every 3 years to every year in order to provide timely health statistics for monitoring changes in health risk factors and diseases and developing associated public health policies and health programmes.^1^ The survey period before 2007 covers 2–3 months of the concerned year, but since then it has covered all weeks of the year, which should solve the problem of seasonal variations.

The 2011 KNHANES response rate was 76.1% for the health interview and examination survey and 82.4% for the nutrition survey; 8518 of 10 589 sampled persons participated in at least one of the three components (i.e. health interview, health examination or nutrition survey).^2^ The sample persons for the nutrition survey were all members of households from which at least one household member participated in the health interview and examination survey.

## Measures

KNHANES collects a number of variables regarding participants’ demographic, social, health and nutritional status from three component surveys: the health interview, health examination and nutrition survey. The health interview and health examination surveys are conducted over 3 days (Wednesday to Friday) for each PSU at MECs, which travel to locations across the country. One unit of MEC consists of two trucks for the exclusive use of KNHANES, which have rooms for surveys and health examination instruments (Figure 1). The nutrition survey is conducted at participants’ homes a week after the health interview. All the surveys are conducted with the participants’ consent. Participants’ consents for additional contacts and the use of unique personal identification numbers are collected for potential follow-up surveys and/or electronic linkage with other secondary data, such as mortality, healthcare utilization and cancer registries.

The health interview questionnaire consists of household and individual components. The household component is based on the information provided by an adult respondent aged 19 years and over from a sampled household and includes variables on demographics on all members of the sampled household and income. The individual component of the health interview questionnaire includes information on cigarette smoking, alcohol use, physical activity, mental health, oral health, weight control and safety, which are collected via self-administration. This individual component questionnaire also collects information on medical conditions, education and occupation, healthcare utilization, activity limitation, quality of life and injury, using a face-to-face interview method. The content of the individual component questionnaire varies with age, considering the different risk factor exposures and disease prevalence (Table 2).

**Table 2:** Survey components of the second round of the fifth Korea National Health and Nutrition Examination Survey in 2011 (KNHANES V-2)

Survey	Components	Method
Health interview	Housing characteristics, medical conditions, socioeconomic status, health care utilization, activity limitation, quality of life, injury	Face-to-face interview in the mobile examination centre (MEC)
	Smoking, alcohol use, physical activity, mental health, oral health, weight control, safety, reproductive health for women	Self- administered in MEC
Health examination	Body measurements, blood pressure, laboratory test (blood and urine), dental measurement, vision, retinal photo and visual field, audiometry, spirometry, balance, bone density and body composition, chest, knee and hip-joint X-ray	Measured and examined in MEC
Nutrition survey	Dietary behaviour, dietary supplement use, food security, food frequency, food and dietary intake	Face-to-face interview in sample person’s home

The health examination collects information regarding obesity, hypertension, chronic obstructive pulmonary disease, eye disease, otolaryngological disease, osteoarthritis and osteoporosis (Table 3). According to standardized protocols, all health examination procedures are performed by trained medical personnel and all equipment is calibrated periodically. Blood and urine samples are collected from participants aged 10 years and over to obtain laboratory results that provide prevalence estimates of diabetes, dyslipidaemia and infectious disease, information on exposure to tobacco smoke and heavy metals, kidney function and thyroid function (added in 2013) (Table 4). In addition, specimens (serum, plasma, DNA) of participants are stored for future genetic research. The laboratory data quality control programme monitors laboratory performance to ensure that all analytical values meet acceptable standards of precision and accuracy. For example, lipid profiles have been compared with those of the US CDC Lipid Standardization Program since 2009. Changes have been made to survey items partly due to the availability of survey resources. Vision and ophthalmology (retinal photos, visual fields), audiometry, balance and dual energy X-ray absorptiometry (body composition, bone density) were included in 2008, and joint X-ray (knee, hip, lumbar) was added in 2009, collaborating with academic societies providing technical advice and highly trained medical personnel.

**Table 3:** Health examination components of the Korea National Health and Nutrition Examination Survey (KNHANES), 1998–2012

Health examination components	Age	'98	'01	'05	'07	'08	'09	'10	'11	'12
Blood pressure	10+ years	○^c^	○	○	○	○	○	○	○	○
Anthropometric measurements	1+ year									
Height		○	○	○	○	○	○	○	○	○
Weight		○	○	○	○	○	○	○	○	○
Waist circumference		○	○	○	○	○	○	○	○	○
Dental examination	1+ year									
Caries		○			○	○	○	○	○	○
Sealants					○	○	○	○	○	○
Periodontal disease		○			○	○	○	○	○	○
Tooth retention					○	○	○	○	○	○
Denture					○	○	○	○	○	○
Spirometry	40+ years									
Restrictive pulmonary disease			○		○	○	○	○	○	○
Obstructive pulmonary disease			○		○	○	○	○	○	○
Chest X-ray	15+ years									
Tuberculosis						○	○	○	○	○
Pneumonia						○	○	○	○	○
Emphysema						○	○	○	○	○
Pneumothorax						○	○	○	○	○
Dual-energy X-ray absorptiometry^a^^,^^b^	10+ years									
Bone density						○	○	○	○	
Body composition						○	○	○	○	
Vision	5+ years					○	○	○	○	○
Opthalmology	19+ years									
Retinal photos						○	○	○	○	○
Visual fields						○	○	○	○	○
Hearing test	12+ years					○	○	○	○	○
Balance	40+ years					○	○	○	○	○
Joint X-ray	50+ years									
Knee							○	○	○	○
Hip							○	○	○	○
Lumbar								○	○	○

^a^Conducted from July 2008 to June 2011.

^b^Conducted in participants aged 19 years and over from July 2008 to June 2009.

^c^○ conducted on concerned year.

**Table 4:** Laboratory test components of the Korea National Health and Nutrition Examination Survey (KNHANES), 1998–2012

Laboratory test components	Age	'98	'01	'05	'07	'08	'09	'10	'11	'12
Dyslipidaemia	10+ years									
Cholesterol(total)		○^d^	○	○	○	○	○	○	○	○
High density lipoprotein(HDL)		○	○	○	○	○	○	○	○	○
Low density lipoprotein(LDL)		○	○	○	○	○	○	○	○	○
Triglycerides		○	○	○	○	○	○	○	○	○
Diabetes	10+ years									
Glucose(fasting)		○	○	○	○	○	○	○	○	○
Glycohaemoglobin		○	○	○	○	○	○	○	○	○
Insulin					○	○	○	○		
Hepatitis	10+ years									
SGOT^a^		○	○	○	○	○	○	○	○	○
SGPT		○	○	○	○	○	○	○	○	○
γ-GTP								○	○	
Hepatitis HBs antigen		○	○	○	○	○	○	○	○	○
Hepatitis C antibody										○
Hepatitis C RNA										○
Metals^b^	10+ years^c^									
Mercury				○	○	○	○	○	○	○
Lead				○	○	○	○	○	○	○
Cadmium				○	○	○	○	○	○	○
Manganese					○	○	○			
Arsenic					○	○	○			
Zinc								○		
Anaemia	10+ years									
Haemoglobin		○	○	○	○	○	○	○	○	○
Haematocrit		○	○	○	○	○	○	○	○	○
Ferritin					○	○	○	○	○	○
RBC		○		○	○	○	○	○	○	○
WBC		○	○	○	○	○	○	○	○	○
Iron								○	○	○
TIBC								○	○	○
Kidney function	10+ years									
BUN		○	○	○	○	○	○	○	○	○
Creatinine		○	○	○	○	○	○	○	○	○
Bone density	10+ years									
ALP						○	○	○	○	
25 hydroxy vitamin D_3_					○	○	○	○	○	○
PTH						○	○	○	○	
Allergy^b^	10+ years									
Total IgE								○		
Specific IgE								○		
Urine test	10+ years									
Protein		○	○	○	○	○	○	○	○	○
Glucose		○	○	○	○	○	○	○	○	○
Blood		○	○	○	○	○	○	○	○	○
Creatinine					○	○	○	○	○	○
Ketone		○		○	○	○	○	○	○	○
Bilirubin		○		○	○	○	○	○	○	○
Gravity		○		○	○	○	○	○	○	○
pH		○		○	○	○	○	○	○	○
Nitrite		○		○	○	○	○	○	○	○
Microalbumin									○	○
Cotinine				○	○	○	○	○	○	
Sodium						○	○	○	○	
Urobilinogen		○	○	○	○	○	○	○	○	○

^a^SGOT, serum glutamate oxaloacetate transaminase; SGPT, serum glutamate pyruvate transaminase; TIBC, total iron binding capacity; BUN, blood urine nitrogen; ALP, alkaline phosphatase; PTH, parathyroid hormone.

^b^Conducted in 1/3 participants.

^c^Conducted in participants aged 20 years and over in 2005, 2007, 2008 and 2009.

^d^○ conducted on concerned year.

The nutrition survey addresses dietary behaviours, food frequency and food intake and is conducted using the face-to-face interview method. The dietary behaviour questionnaire includes meal skipping, eating out, eating with family, taking dietary supplements, nutrition education, use of food labelling and food security. The food frequency questionnaire is composed of 63 food items that are key sources of energy and nutrients. The food intake questionnaire has been designed as an open-ended survey for reporting various dishes and foods using the 24-h recall method with various measuring aids.

The survey staff members are required to complete an intensive training course and to conduct supervised practice before working in the survey field. Retraining sessions are provided five to six times a year to reinforce the proper protocols and techniques. In addition, 30 expert committees composed of over 120 experts (usually nominated by the associated academic societies in Korea) have technically supported KNHANES regarding quality assurance and control of the survey and the selection of individual survey items (e.g. smoking, alcohol etc.). Detailed quality control instructions based on the consensus of committee members have been implemented in the survey and are described in the survey manuals.

## Data resource use

KNHANES data are widely used by governmental organizations and researchers. The Korean Government has periodically revised national health plans and recently established the National Health Plan 2020 (HP 2020).^3^ KNHANES has supported health statistics on more than half of target indicators for HP 2020 goals. KNHANES has been used for the development of Korean standards regarding health and nutrition, such as the Dietary Reference Intakes for Koreans,^4^ and will be used for the 2017 Growth Charts for Korean infants, children and adolescents. The data were also used in international comparison studies. Recent publications on inequalities in non-communicable diseases and the global burden of metabolic risk factors used several rounds of KNHANES data and provided comparable health statistics.^5–7^

**Figure 2:**
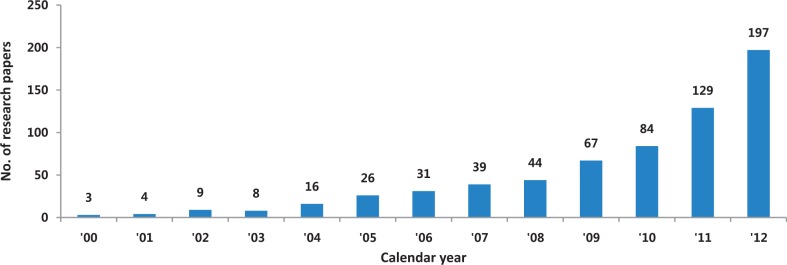
Trends in the number of research papers using the Korea National Health and Nutrition Examination Survey (KNHANES) data, results from electronic search on 26 March 2013 in Pubmed and Korea Med

A growing number of research papers have used KNHANES data (Figure 2). Based on the reports to KCDC, at least 500 publications mainly appearing in domestic and international journals have been listed on KNHANES website (http://knhanes.cdc.go.kr). A list of English-language papers using KNHANES data is available as Supplementary data at *IJE* online. The main research issues are changes in health problems and nutritional status and the association between the prevalence and risk factors of chronic diseases. For example, there are recent reports on the nutritional transition in Korea during the past 10 years^8^ and the changes in the prevalence and control rate of hypertension among Korean adults diagnosed with diabetes.^9^ Groups of researchers studied the determinants of serum parathyroid hormone and bone mineral density in the low calcium-intake population^10^ and the association between quality of life and the amount and pattern of chronic disease in the elderly population.^11^ KCDC has published articles on data processing protocol^12^ and reports on analysis results using the data, including the national prevalence of chronic diseases such as otolaryngological diseases, chronic obstructive pulmonary diseases and ophthalmic diseases.^13–16^

KCDC has provided annual workshops for data users to promote the broader and more proficient use of the data. Between 2011 and 2012, the workshops were held three times, with 400–800 researchers in Korea attending each workshop (see (B) in Figure 1).

## Strengths and weaknesses

KNHANES is a continuous survey with nationally representative samples of Korea, and the health interview, physical examination and nutrition survey are combined to assess associations between variables. KNHANES data are valuable sources for monitoring changes in risk factors and diseases and identifying target groups in need of interventions. KCDC and related academic societies have managed external quality control programmes for all steps (including survey administration, data collection, laboratory analysis and data processing) as well as internal quality assurance and control procedures. Because the survey components and methods could partly vary with the year, data users should be cautious of the changes in the detailed survey methods and questionnaires of concerned variables, especially in studies on secular trends, and should refer to survey manuals thoroughly.

## Data resource access

The reports and microdata of KNHANES are released annually (see (A) in Figure 1). The data of the respective year are available to the public at the end of the following year free of charge. KCDC has also published documents on survey manuals and presented primary results through the official website of KNHANES (http://knhanes.cdc.go.kr). KCDC is now preparing an English-language information homepage, which will be available by the end of 2014. Further information and enquiries can be submitted to the corresponding author, K. Oh, at kwoh27@korea.kr.

## Supplementary Data

Supplementary data are available at *IJE* online.

## Funding

KNHANES has been financially supported by the Health Promotion Fund of Korea with administrative support from the Ministry of Health and Welfare. Furthermore, this surveillance system is technically supported by major academic societies in Korea based on the memoranda of understanding between KCDC and those academic societies.

## Supplementary Material

Supplementary Data
