# Supplementary material for: Data Resource Profile: The Korea National Health and Nutrition Examination Survey (KNHANES)
Source: Int J Epidemiol. 2014 Feb 27;43(1):69–77. doi: 10.1093/ije/dyt228 (PMC3937975; doi:10.1093/ije/dyt228)
Supplement: Supplementary Data [file supp_43_1_69__index.html]

Supplementary Data 

# Data Resource Profile: The Korea National Health and Nutrition Examination Survey (KNHANES)

## Supplementary Data

files

**Files in this Data Supplement:**

- Supplementary Data - xlsx file
